# Anticancer Effects of Arsenic Compounds in Non-Small Cell Lung Cancer

**DOI:** 10.54457/dr.202402003

**Published:** 2024-09-20

**Authors:** Neelima Konduri, Anita Thyagarajan, Ravi P. Sahu

**Affiliations:** 1 Department of Pharmacology and Toxicology, Boonshoft School of Medicine Wright State University, Dayton, OH 45435, USA

**Keywords:** Non-small cell lung cancer, Chemotherapy, Drug repurposing, Arsenic compounds, Cell signaling pathways

## Abstract

Non-small cell lung cancer (NSCLC) is the most common and prevalent subtype of lung cancer and continues to be one of the leading causes of cancer-related deaths worldwide. Despite various treatment options, a majority of NSCLC patients continue to experience disease progression and associated side effects, which are largely attributed to drug resistance, indicating the need for alternative strategies to combat this deadly disease. Among various applicable alternative approaches, repurposed drugs such as arsenic compounds have been shown to exert anticarcinogenic properties against NSCLC and possess the ability to overcome drug resistance mechanisms. Notably, numerous studies have demonstrated that the antitumor effects of arsenic compounds such as arsenic trioxide, arsenic sulfide, and tetra arsenic hexoxide are mediated via their ability to target several oncogenic signaling pathways, including nuclear factor-kappa B (NF-kB), epidermal growth factor receptor (EGFR), and signal transducer and activator of transcription 3 (STAT3). Inhibition of such signaling cascades results in altered cellular activities, including cell cycle arrest, decreased proliferation, and increased apoptosis. Importantly, these arsenic compounds have also been shown to overcome tumor resistance mechanisms and/or exert synergy in combination with other therapeutic agents resulting in the augmentation of cancer cell cytotoxicity. This review highlights the anticarcinogenic mechanisms of arsenic compounds and their impact on the efficacy of therapeutic agents.

## Introduction

Lung cancer is one of the most prevalent malignancies and 70% of lung cancer patients experience locally advanced tumors at the time of diagnosis^[1–4]^. Of two major types, including small cell lung cancer (SCLC), non-small cell lung cancer (NSCLC) is the most prevalent which accounts for 80–85% of lung cancer cases and is associated with the highest mortality rates^[4,5]^. Notably, adenocarcinoma, squamous, and large cell carcinomas are three subtypes of NSCLC ^[4,5]^. According to the American Cancer Society, 117,910 men and 118,830 women will die from lung cancer in the United States in 2023, with an estimated 238,340 new cases and 127,070 fatalities (67,160 in men and 59,910 in women)^[3]^. It is important to note that since 1987, lung cancer has been linked to more female fatalities than breast cancer^[1,2,4,5]^. Notably, smoking, genetic predisposition, aging, air pollution, and mixed-profession exposures, including radiation, and family history are the major risk factors associated with NSCLC^[4–6]^.

Importantly, the patient’s condition, tumor stage, and the molecular characteristics of NSCLC all influence the course of treatment, which includes surgery, radiotherapy, chemotherapy, immunological therapy, and targeted therapy^[5–9]^. As a result of technological developments, over the past few decades, radiotherapy has become much more precise and tolerable^[10–12]^. Besides this, immunotherapy approaches, particularly immune checkpoint inhibitors (ICI) that target programmed death receptor 1 (PD-1), its ligands, PD-L1/L2, and cytotoxic T-lymphocyte-associated protein 4 (CTLA-4) have shown promising outcomes for treating NSCLC patients^[7,13–15]^. However, when single-agent immunotherapy or combination immunotherapy is not an option for patients, chemotherapy regimens are considered their first choice of treatment^[7,11,16]^. Despite conventional therapies, targeted therapy, commonly referred to as “molecular targeted therapy,” that targets specific molecules such as altered proteins or signaling pathways in cancer cells, is currently been used to treat NSCLC patients. This includes the protein receptor tyrosine kinase (ROS1), the anaplastic lymphoma kinase (ALK) protein, and the epidermal growth factor receptor (EGFR)^[7,16–18]^. Notably, radiofrequency ablation (RFA) is used as another good alternative for NSCLC^[19–22]^.

While most viable treatment options, including chemotherapy, targeted therapy, and immunotherapy exert promising antitumor effects at initial stages, their effectiveness in the long-term is often hampered via counter-regulatory mechanisms^[23–30]^, indicating the need for the exploration of underlying mechanisms of drug resistance to identify new targets or novel combination approaches. To that end, the aberrant activation of mesenchymalepithelial transition tyrosine kinase receptor (MET or c-MET) and hepatocyte growth factor (HGF) is involved in the development of human malignancies, including lung cancer^[31,32]^. Therefore, the development of small molecular tyrosine kinase inhibitor (TKI), crizotinib that targets ALK, c-MET/hepatocyte growth factor receptor (HGFR), and ROS1 has been shown to possess superior activity, including longer progression-free survival (PFS) than chemotherapeutic regimens in previously untreated NSCLC patients^[32–34]^. However, its therapeutic efficacy was compromised in patients who received prior chemotherapy^[33,34]^, indicating that chemotherapy-resistant NSCLC patients exhibit poor efficacy of crizotinib despite having altered/aberrant activation of known targets, ALK, c-MET/HGFR, or ROS1, suggesting the exploration of alternative treatment options.

Along similar lines, a combination of chemotherapy or antibody-drug conjugates (ADCs) with radiation therapy (RT) has been shown to enhance cancer cell sensitivity or therapy effectiveness for solid tumors, including NSCLC^[35]^. However, non-cancerous tissue has also been documented to result in enhanced RT sensitivity or associated off-target toxicity, resulting in adverse effects or discomfort for patients compared to RT treatment alone^[35–37]^. Of significance, diminished responses of ICI or ADCs have been documented in a majority of NSCLC patients having driver mutations or alterations in oncogenes, including EGFR, ALK, ROS1^[37,38]^. This indicates that genomic testing of tumor samples for driver mutations/genetic alterations could rationalize prospective treatment strategies for such NSCLC patients. Importantly, the efficacy of some arsenic compounds such as As_4_S_4_ has been documented in reversing cisplatin chemotherapy resistance in NSCLC cells^[39]^. This provides the rationale for a promising alternative drug repurposing approach for combination therapy, including with ADCs or ICI in overcoming drug resistance mechanisms and increasing their therapeutic synergy.

## Arsenic compounds

Arsenic is a naturally occurring chemical element that is widely dispersed in the earth’s crust and can be broadly divided into inorganic and organic compounds^[16,40,41]^. While arsenic has traditionally been considered a toxic agent, it has ironically been employed as a therapeutic agent to treat a variety of illnesses and is known to have antibacterial, antiviral, antiparasitic, and anticancer properties^[40,42,43]^. While most arsenic-based medications are no longer in practice, some of them were explored because of their promising therapeutic indications. Importantly, acute promyelocytic leukemia and several other malignancies like breast, pancreatic, and gastric cancers were proven to be dramatically managed by arsenic trioxide, the primary component of traditional Chinese medicine^[40,42,44–46]^. In addition, other abnormalities, including, dyspepsia, anemia, and rheumatism are also reported to be treated by arsenic trioxide^[43,45]^.

Notably, arsenic compounds have also been considered due to their abilities to target numerous signaling molecules, including embryonic stem cell transcription factors such as Oct4, and SOX2, vascular endothelial growth factor (VEGF), hypoxia-inducible factor-1 alpha (HIF-1α), VEGF receptor 2 (VEGFR-2), Dll4, Notch-1, GS-X pumps and ABC transporters that not only regulate the growth/activities of tumor cells, including cell cycle disruption and angiogenesis, but also involved in reduced efficacy of therapeutic agents^[47–49]^. Besides arsenic compounds-induced apoptosis is mediated via their ability to activate the tumor necrosis factor-related apoptosis-inducing ligand (TRAIL) and FAS receptors^[24,46,50]^. While immunotherapy demonstrates promising outcomes in certain NSCLC patients, it is associated with tolerance and low effectiveness. Thus, arsenic compounds could provide alternative options to those individuals who are sensitive to immunotherapy due to their potential to regulate and interrupt several growth enhancing mechanisms^[39]^. Furthermore, arsenic compounds have demonstrated to improve the effectiveness of chemotherapeutic agents and targeted therapy such as cisplatin and gefitinib, indicating their potential to be explored as a promising approach for multifaceted intervention for managing NSCLC^[19,51]^. As a result, the implication of arsenic compounds on the clinical application has also been an important area of investigation and has attracted attention.

**Fig. 1.**
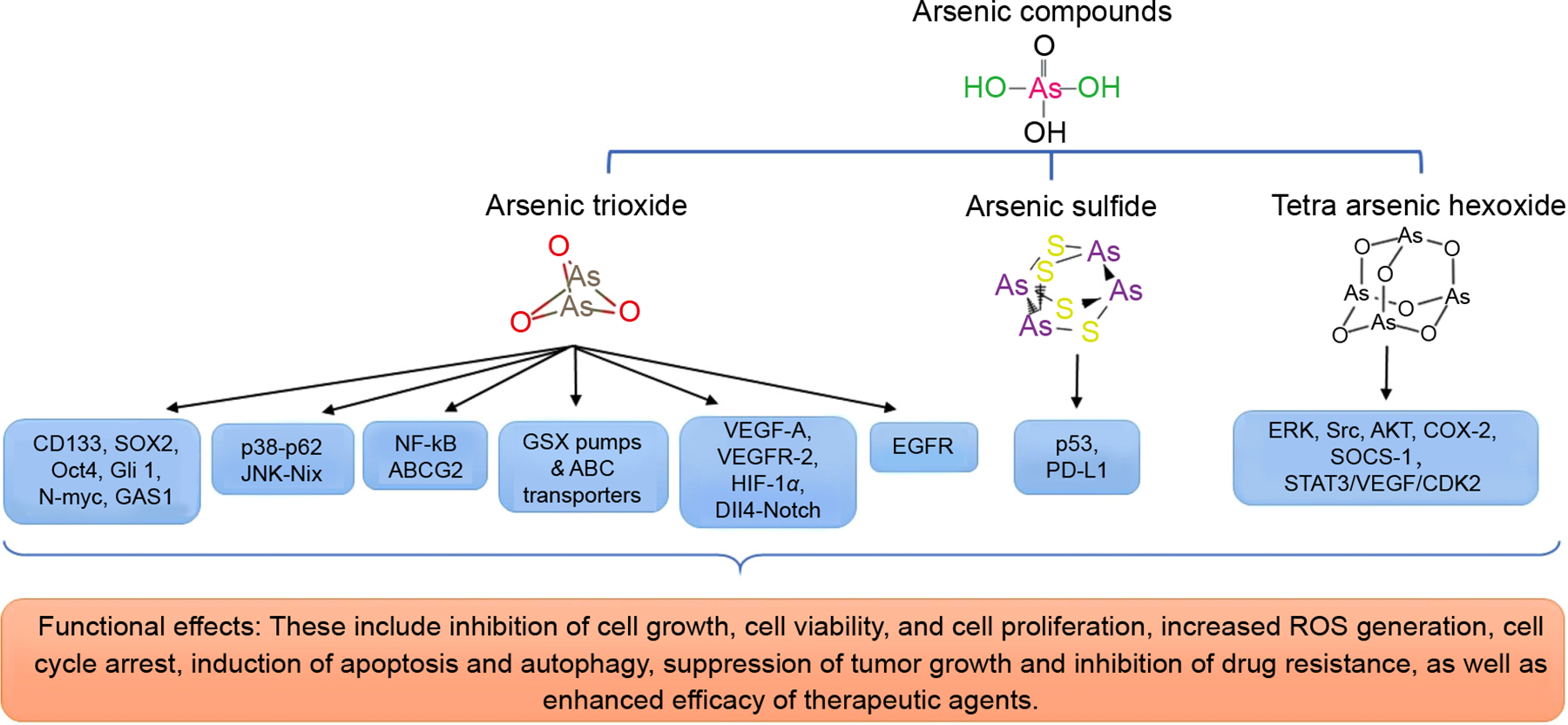
The structures of arsenic trioxide, arsenic sulfide, and tetra arsenic hexoxide and their targeted signaling pathways are shown. 1. CD133: Cluster of Differentiation 133 (also known as Prominin-1). 2. SOX2: SRY (sex determining region Y)-box 2. 3. OCT4: Octamer-binding Transcription Factor 4 (also known as POU5F1). 4. Gli1: Glioma-associated oncogene-1. 5. N-myc: It is a proto-oncogene protein (a member of the MYC family of transcription factors). 6. GAS1: Growth Arrest-Specific 1 7. p38-p62: p38 is a mitogen-activated protein kinase-38, and p62 stands for sequestosome 1 (SQSTM1), commonly referred to as p62. 8. Nix-JNK: Nix is a NIP3-like protein X (also known as BNIP3L), and JNK is Janus kinase pathway. 9. NF-kB: It is Nuclear Factor Kappa-light-chain-enhancer of Activated B Cells. 10. ABCG2: It is ATP (Adenosine Tri Phosphate)-Binding Cassette Subfamily G Member 2 11. GSX pumps: Glutamate Sodium Exchange Pumps. 12. VEGF-A: Vascular Endothelial Growth Factor A. 13. VEGFR2: Vascular Endothelial Growth Factor Receptor 2. 14. HIF-1α: Hypoxia-Inducible Factor 1-alpha. 15. DII4: Delta-like 4 (Dll4) - Notch Signaling Pathway. 16. EGFR: Epidermal Growth Factor Receptor. 17. p53: Tumor suppressor protein 53 18. PDL-1: Programmed Death-Ligand 1 19. ERK- Extracellular Signal-Regulated Kinase. 20. Src: It is a signaling pathway that involves the Src family of protein tyrosine kinases. 21. AKT: Serine threonine-specific protein kinases 22. SOCS-1: Suppressor of Cytokine Signaling 1 23. STAT3: Signal Transducer and Activator of Transcription 3 24. CDK2: Cyclin-Dependent Kinase 2 25. Arsenic trioxide- As_2_O_3_ 26. Arsenic sulfide -As_4_S_4_ 27. Tetra Arsenic hexoxide-As_4_O_6_.

Importantly, given that the ongoing treatment options are associated with the development of tumor resistance mechanisms, other approaches such as drug repurposing with anticancer properties, including arsenic compounds are being explored for cancer treatment. To that end, arsenic compounds such as arsenic trioxide represent a novel strategy for the treatment of both drug-resistant and advanced-stage NSCLC^[15,19,26]^. Notably, the inorganic arsenicals such as arsenic trioxide, arsenic sulfide, and tetra arsenic hexoxide also possess promising anticancer properties. This article highlights the mechanisms and therapeutic potential of inorganic and organic arsenic, particularly, arsenic trioxide, arsenic sulfide, and tetra arsenic hexoxide. The structure and mechanisms by which these arsenic compounds exert anti-cancer activities are shown in Fig. 1 and summarized below.

## Arsenic compounds as promising therapeutic agents

Arsenic trioxide as a potential drug repurposing candidate for NSCLC

Previous studies have demonstrated that arsenic trioxide (As_2_O_3_) is a promising candidate to be explored as a repurposed drug, causing remission of acute promyelocytic leukemia (APL) with little to no toxicity, and also significantly reduces the ability of APL stem cells to self-renew^[48]^. Considering its promising effect on APL, a study led by Ke-Jie Chang and colleagues evaluated the effect of As_2_O_3_ on two different cancer stem cells (CSCs), such as NCI-H460 (NSCLC stem cells) and NCI-H446 cells (SCLC stem cells), which are implicated in chemotherapy resistance and tumor recurrence. The colony formation assay revealed that As_2_O_3_ could remarkably inhibit the colonogenic capacity of NSCLC with higher effects noted in SCLC stem cells. The tumorsphere assay showed that As_2_O_3_ was not only able to significantly decrease the number but also the size of tumorspheres. Notably, As_2_O_3_ treatment dramatically decreased the mRNA and protein levels of CD133, a CSC marker in a dose-dependent manner. Moreover, the levels of SRY-box transcription factor 2 (SOX2) and octamer-binding transcription factor 4 (Oct4), the two transcription factors crucial for controlling the self-renewal and multipotency of CSCs were also decreased by As_2_O_3_ treatment. Furthermore, the investigation of underlying mechanisms of As_2_O_3_’s inhibitory effects revealed its ability to block the oncogene and a critical transcriptional factor called glioma-associated oncogene-1 (Gli1), as well as downregulate the N-myc proto-oncogene protein and growth arrest specific-1 gene (GAS1), which activate the hedgehog signaling pathway. Overall, these findings indicated novel mechanisms by which As_2_O_3_ inhibits the growth of NSCLC^[48]^. The summary of in vitro and/or in vivo studies is given in Table 1. We first highlighted the in vitro studies with As_2_O_3_ then its combination with signaling cascade inhibitor(s) or chemotherapy agent followed by studies where both in vitro and in vivo models were used, and then studies with other arsenic compounds such as As_4_S_4_.

Along similar lines, Lee *et al*., and colleagues evaluated the underlying mechanisms of sodium arsenite (NaAsO_2_) sensitivity in the NCI-H460 NSCLC cell line. It was found that NaAsO_2_ treatment reduced the cell viability in a dose-dependent manner. To determine the mechanisms involved in NaAsO_2_-induced decreased cell viability, the levels of autophagy marker LC3-II and the monomeric and multiple HMW (high molecular weight) forms of p62 protein were accessed. The data demonstrated that LC3-II and p62 levels were initially elevated but decreased at higher NaAsO_2_ concentrations. Additionally, the ability of NaAsO_2_ to induce mitophagy was examined using the monomeric and HMW forms of the mitophagy receptor Nix as a marker. It was discovered that NaAsO_2_ exposure increases the expression of both forms of Nix in a dose-dependent manner. To determine whether NaAsO_2_ cytotoxicity was because of the induction of apoptosis, the apoptosis-related proteins procaspase-8, procaspase-3, and PARP-1 were investigated. It was found that NaAsO_2_ treatment induced cleavage of these apoptosis proteins. Besides, NaAsO_2_ exposure also contributed to Bax cleavage and its time-dependent localization to mitochondria, resulting in mitochondrial damage. Moreover, the studies with p62 and Nix knockdowns were performed to examine their roles in autophagy and apoptosis induction. Although p62 knockdown resulted in NaAsO_2_-induced increased levels of LC3-II and Nix expression, Nix knockdown did not affect these protein levels. These results indicated that transient autophagic degradation was inhibited by NaAsO_2_ which promoted Nix accumulation via modulating p62 expression. Furthermore, activation of the mitogen-activated protein kinases (MAPK) family proteins such as c-Jun N-terminal kinase (JNK), and p38 signaling pathways were found to be involved in NaAsO_2_-induced cytotoxic effects. Consequently, significantly increased reactive oxygen species (ROS) production in NaAsO_2_-exposed NCI-H460 cells was found and its level was attenuated by the antioxidant N-acetylcysteine (NAC). In addition, JNK and p38 pathways were also suppressed by NAC treatment. Overall, these findings indicate that p38-p62 and JNK-Nix signaling pathways were involved in inducing arsenic sensitivity in NSCLC^[49]^.

Of significance, adenosine triphosphate (ATP)-binding cassette (ABC) transporter family members such as ABCB1, ABCC1, and ABCG2 have been shown to induce resistance to As_2_O_3_ via mechanisms involving the efflux of tri gamma-glutamyl cysteinyl glycine thioarsenite (AsIII(GS)3) complex^[52–57]^. To that end, a study by Jiang and colleagues investigated the mechanisms of ABC transporters-induced drug resistance in affecting the growth of A549 NSCLC cells in response to As_2_O_3_ treatment. The first studies demonstrated that ABCG2 is involved in arsenic complex [AsIII(GS)3] extradition in A549 cells as a significantly higher increase in the levels of ABCG2 was noticed upon As_2_O_3_ treatment compared to the other two transporters, ABCB1 and ABCC1. To comprehend the molecular pathways underlying increased ABCG2 expression in As_2_O_3_- treated cells, the role of the nuclear factor kappa B (NF-κB) signaling was evaluated. Immunofluorescence studies revealed that the upregulation of ABCG1 was due to the activation of the NF-κB pathway by As_2_O_3_. To validate NF-κB activation by As_2_O_3_, A549 cells were treated with novobiocin (NF-κB inhibitor), pyrrolidine dithiocarbamate (PDTC, NF-κB antagonist), and tumor necrosis factor α (TNF-α, NF-κB agonist) with As_2_O_3_. The data demonstrated that As_2_O_3_ upregulates and PDTC downregulates NF-kB activation and that higher NF-kB expression was observed with a combination of As_2_O_3_ and TNF-α treatment. Moreover, an optimal concentration of novobiocin and PDTC both potentiated the cytotoxic effect of As_2_O_3_ on A549 cell viability. Furthermore, compared to novobiocin, PDTC significantly increased apoptosis, whereas TNF-α exhibited the opposite effect. These findings indicated that the suppression of NF-κB/ABCG2 axis represents a new approach for the treatment of NSCLC^[58]^.

Although cisplatin (CDDP) is a typical component of combination chemotherapy and a promising anticancer agent, it often develops resistance in a variety of malignancies. To explore the strategy for overcoming CDDP resistance, a study conducted by Toshihiro Suzuki and colleagues investigated As_2_O_3_ effects using CDDP-resistant PC-9/CDDP and CDDP-sensitive PC-14/CDDP cell lines. The data demonstrated that PC-9/CDDP cells exhibited hypersensitivity to arsenic isoforms such as As_2_O_3_ and arsenate (AsO4). The next studies explored the mechanism of As_2_O_3_ hypersensitivity, and it was found that As_2_O_3_ accumulation was greater in PC-9/CDDP cells compared to parental PC-9 cells. The accumulation or efflux of As_2_O_3_ was mediated by the glutathione S-conjugate export pump (GS-X pump). Furthermore, the roles of the GS-X pump, cellular GSH levels, glutamate-cysteine ligase (γ-GCS), glutathione transferase (GST) activity, and expression of the ABC transporters in downregulating As_2_O_3_ accumulation were explored. It was found that GST activity was significantly lower in PC-9/CDDP cells compared to PC-9 parental cells. In contrast, except for ABCC4, the remaining transporters (ABCC1, 2, and 3) were down-regulated. Moreover, the effect of As_2_O_3_ on CDDP-resistant and CDDP-sensitive cell lines was investigated, which revealed that PC-14/CDDP cells exhibited higher sensitivity to As_2_O_3_ compared to PC-9 and PC-9/CDDP cell lines. Overall, increased As_2_O_3_ sensitivity was found to be mediated via decreased expression of ABC transporters in both CDDP-sensitive parent cells and CDDP-resistant cells^[19]^.

Studies by Yang and colleagues determined the impact of As_2_O_3_ on the progression of SCLC and NSCLC. The in vivo studies utilized A549, NCI-H460, and NCI-H446 cell lines in transplant tumor models and mice were treated for 10 consecutive days with 2.5 mg/kg or 5.0 mg/kg of As_2_O_3_. It was discovered that As_2_O_3_ resulted in greater tumor suppressive activity in NCI-H460 tumor xenograft model where mice treated with 5.0 mg/kg As_2_O_3_ showed significantly reduced tumor growth (similar to a sorafenib-treated group used as a positive control) compared to control group. Besides, tumor suppressive activity of As_2_O_3_ was also documented in NCI-H446 and A549 xenografts. Tumor tissues from As_2_O_3_-treated group exhibited notably reduced levels of cell proliferation marker Ki-67 compared to the control group indicating that As_2_O_3_ could suppress the proliferation of lung malignancy. Importantly, compared to the control group that displayed an increased tumor vascularization, and extended tube-shaped framework, tumors from the As_2_O_3-_treated group showed a noticeably decreased number of blood vessels and vascular networks, as in the sorafenib-treated group, indicating decreased tumor angiogenesis as demonstrated by immunofluorescent CD31 staining. Mechanistically, this As_2_O_3_-induced effect was found to be mediated via reduced levels of VEGF-A and VEGFR-2 expression in all tumor xenografts. Interestingly, in vivo findings of reduced VEGF-A and VEGFR-2 expression were also seen in vitro in A549, NCI-H460, and NCI-H446 cell lines. In addition, As_2_O_3_ treatment in vitro and in vivo resulted in dose-dependent decreased expression of HIF-1α, and Dll4-Notch pathway that are involved in the regulation of tumor angiogenesis. Moreover. in HUVEC cells, As_2_O_3_ dose-dependently suppressed the mRNA and protein expression of VEGFR-2 and Dll4 confirming that As_2_O_3_ inhibits the growth of lung tumor angiogenic activity by reducing the levels of markers related to the VEGF signaling. Overall, these investigations showed that As_2_O_3_’s anti-tumor effect is mediated via its anti-angiogenesis activity involving the inhibition of Dll4-Notch and VEGF signaling networks in both NSCLC and SCLC^[47]^.

Importantly, EGFR amplification is prevalent in almost 60% of NSCLC cases and is correlated with a poor prognosis^[59–62]^. Notably, EGFR belongs to the ErbB family, which is a transmembrane tyrosine kinase receptor, and one of the major oncogenic drivers that is mutated in certain NSCLC cases^[59–62]^. Besides, EGFR mutations are well-known and potential targets for anticancer treatment^[59–62]^. To that end, Jianhua Mao conducted a study to evaluate how EGFR L858R/T790M mutation can be targeted and degraded by As_2_O_3_ treatment using three NSCLC (NCI-H1975, HCC827, and A549) cell lines. The data demonstrated that the proliferation of gefitinib-resistant NCI-H1975 cells harboring EGFR L858R/T790M mutation was markedly reduced by gefitinib and As_2_O_3_ treatment, whereas the impact of this combination on gefitinib sensitive ΔE746-A750 mutant HCC827 and EGFR wildtype A549 cell lines was modest. Additional tyrosine kinase activity testing revealed that NCI-H1975 and HCC827 cell lines exhibited greater calibrated kinase activity (CKA) values than A549 cells, demonstrating an increase in mutation frequency. Notably, even at lower concentrations, gefitinib treatment effectively reduced CKA levels in HCC827 cells. To define the mechanism of decreased cell proliferation, it was found that Z-VADFMK, a caspase inhibitor, and MG132, a proteasome inhibitor, did not prevent As_2_O_3_-mediated EGFR degradation in NCI-H1975 cells. Therefore, the autophagic mechanism was examined, which revealed that As_2_O_3_ increased the expression of LC3-II in NCI-H1975 cells. Besides, the data indicated that As_2_O_3_ targets the EGFR’s functional activity and that P62 mediates As_2_O_3_-induced EGFR degradation. The co-immunoprecipitation assay was used to further assess the impact of As_2_O_3_ on the EGFR-P62 complex. The results demonstrated that EGFR-p62 binding occurs in all three NSCLC cell lines; intriguingly, this binding was found to be strengthened in NCI-H1975 cells harboring EGFR L858R/T790M mutation. Moreover, the in vivo studies demonstrated that the growth of NCI-H1975 and HCC827 xenografts in NOD-SCID mice was inhibited by both As_2_O_3_ and gefitinib combination compared to individual treatments. Overall, the data indicated that As_2_O_3_ targets the EGFR signaling pathway to inhibit both tumor growth and the development of drug resistance in gefitinib-resistant NSCLC cells ^[51]^.

### Arsenic sulfide in reversing cisplatin resistance

Immune cells such as T, B, and myeloid cells express immune checkpoint receptors such as PD-1 on their cell surfaces, which play critical roles in immune control^[59,63,64]^. PD-1 binds to its ligand, PD-L1 and transmits a negative response that encourages T-cell tolerance and immunity, resulting in reducing CD8 + T-cell survivability and effector activity^[59,63,64]^. Thus, autoimmune disorders can be brought on by PD-1 imbalances and deficiencies^[59,63,64]^. To evade antitumor immune responses and hinder the immune system from destroying cancer cells, PD-L1 expression in tumor cells activates the immunological checkpoint PD-1/PD-L1 axis. As a result, PD-1/PDL-1 axis-targeting immune checkpoint inhibitors (ICIs) were introduced as a cancer treatment. Arsenic sulfide (As_4_S_4_) is another inorganic arsenic compound that exhibits antitumorigenic effects and a study aimed at evaluating As_4_S_4_ effects on PDL-1 expression, its roles in cisplatin-resistant A549/DDP, H1299/DDP and their parental A549, H1299 cell lines, as well as reversing cisplatin drug resistance^[39]^.

The first studies demonstrated that PD-L1 expression was higher in A549/DDP and H1299/DDP cell lines compared to their parental cell lines, Next studies determined the underlying mechanisms involved in the upregulation of PD-L1 expression, and the data revealed that interferon- γ (IFN-γ) treatment increased PD-L1 protein expression even in the parental A549 and H1299 cell lines in a dose-dependent manner, and when co-treated with cisplatin (DDP), increased DDP resistance was noticed. To overcome DDP resistance, PD-L1 was silenced in both the A549 and H1299 cell lines, which increased DDP sensitivity, indicating that PD-L1 ablation abrogates DDP resistance^[39]^. Moreover, to investigate whether As_4_S_4_ facilitates the sensitivity of A549/DDP and H1299/DDP to DDP synergistically, these cell lines were treated with As_4_S_4_ along with DDP. It was found that As_4_S_4_ and DDP + As_4_S_4_ enhanced the sensitivity of A549/DDP cells to DDP and the IC_50_ values were decreased upon co-treatment when compared to DDP-alone treated A549/DDP and H1299/DDP cell lines. In addition, As_4_S_4_ also enhanced DDP-induced apoptosis in A549/DDP cells and downregulated PD-L1 expression by upregulating p53 and miR-34a-5p expression in a time-dependent manner whereas As_4_S_4_ exhibited no effect in H1299/DDP cells. In contrast, the miR-34a-3p levels were unaltered in both the DDP-resistant cell lines A549/DDP and H1299/DDP^[39]^.

To examine the p53 function in PD-L1 regulation and determine whether p53 inhibition may affect PD-L1 expression, A549/DDP cells were treated with As_4_S_4_ in the presence and absence of PFT-α (a p53-specific inhibitor). It was found that PFT-α decreased p53 and miR-34a-5p levels and suppressed apoptosis in combination with As_4_S_4_ and DDP in A549/DDP cells. In in vivo studies, A549/DDP cells were subcutaneously implanted into BALB/c nude mice and treated with or without DDP and As_4_S_4_. The inhibition of tumor development was significantly higher with the combination of As_4_S_4_ and DDP compared to individual As_4_S_4_ and DDP treatments. Besides, the p53 expression was significantly increased with As_4_S_4_ treatment and downregulated DDP induced-PD-L1 upregulation. Overall, the studies indicated that cisplatin resistance in NSCLC can be reversed by As_4_S_4_ and that PD-L1 is a potential target for reversing DDP resistance^[39]^. However, given the heterogeneity of cancer cells, such evidence needs to be validated on additional cell lines, and possibly, on primary specimens.

### Tetra arsenic hexoxide and combination of antitumor herbal agents

Apart from As_2_O_3_ and As_4_S_4_, tetra arsenic hexoxide (As_4_O_6_) has been shown to exhibit greater antitumor effects in malignancies like cervical and nasopharyngeal squamous cancers^[50,65,66]^. Moreover, As_4_O_6_ in combination with other natural antitumor agents has been reported to enhance NSCLC cell cytotoxicity^[50]^. Along similar lines, a study aimed to investigate the As_4_O_6_ effect in NSCLC cells in combination with an herbal mixture having natural antitumor agents, Oldenlandia diffusa and Salvia miltiorrhiza along with their ethanolic extracts (termed as OS). Besides, the effect of a combination of PR (arsenic herbal mixture) and OS (ethanol extract of natural antitumor agents) (i.e., PROS) was evaluated. It was found that PROS exhibited greater cytotoxic effects in both A549 and NH460 NSCLC cell lines when compared to PR or OS alone. Notably, except for oral squamous cells (YD-8), a similar effect was exerted by PR and OS in the colon (HCT-116), renal (786-O), and prostate (PC-3 & DU145) cancer cell lines^[50]^.

Furthermore, the activation of STAT3 was examined and found to be low in YD-8 cells as compared to other cell lines and only PROS treatment resulted in apoptosis induction compared to monotherapy. From the cell cycle analysis, it was confirmed that apoptotic bodies were elevated in both NSCLC cells whereas G1-sub population enhancement and S-phase arrest by PROS were seen only in A549 cells compared to PR or OS alone treatments. Besides, PROS downregulated the antiapoptotic Bcl-2 protein, activation of ERK, Src, and AKT as well as cell cycle regulating genes (Cyclin E, Cyclin A, CDK2), E2F1, IL-6, COX-2, and SOCS-1, and impaired STAT3 ability to bind with CDK2 and VEGF in H1299, A549, H460 NSCLC cell lines^[50]^. Using HUVEC cells, it was found that PROS inhibited angiogenesis by blocking the activation of VEGFR2/SRC/STAT3 axis. In vivo studies were conducted with H460 cells to confirm the apoptotic and antiangiogenic effects of PROS, which revealed significantly decreased tumor growth was mediated via the activation of cleaved caspase 3 and downregulation of VEGF and STAT3 expression as compared to the control group. Overall, these findings indicated that PROS acts as an effective anticancer agent for NSCLC via its ability to exhibit both antiangiogenic and apoptotic effects mediated via the inhibition of STAT3/VEGF/CDK2 signaling pathway^[50]^.

### Effects of long-term exposure to arsenic

Although previous studies showed that arsenic is a potential drug repurposing agent for NSCLC, other studies showed that long-term exposure to arsenic exerts undesired effects and can lead to cancer progression via the involvement of multiple pathways, including the EGFR signaling^[67–70]^. While EGFR plays a crucial role in developmental biology, tissue homeostasis, and wound healing, its overexpression or hyperactivation along with other EGFR family members (i.e., ErbB2, ErbB3, and ErbB4) is used as targets in a variety of cancers.

On a similar line, a study evaluated the impact of acute and chronic exposure to arsenic on EGFR signaling. To that end, human lung epithelial BEAS-2B cells were exposed to acute arsenic (5 μM for 24 hours), and chronic low level (100 nM for 24 weeks) sodium arsenite (NaAsO_2_, termed as arsenic). It was found that acute arsenic exposure resulted in increased EGFR expression and decreased cell viability. While chronic arsenic exposure did not affect cell viability, it increased EGFR expression and activation (phosphorylation) and protein levels of transforming growth factor-α (TGFα) as well as resulted in EGFR ligand (i.e., EGF)-independent enhanced cell migration. To evaluate whether TGFα and EGF exert distinct effects in BEAS-2B cells, naive BEAS-2B cells were treated with EGF or TGFα, and it was found that both these treatments downregulated EGFR; however, its level was amplified within the prolonged low dose chronic arsenic treatment. The data revealed that chronic arsenic (100 nM) exposure elevated TGFα production which further enhanced the EGFR protein expression. Overall, these findings revealed that chronic arsenic exposure of BEAS-2B cells enhances TGFα expression, cell migratory ability, EGFR phosphorylation and its cell surface expression, which are distinctive from acute arsenic-mediated effects^[69]^. The summary of studies using chronic (long-term) arsenic exposure is given in Table 2.

Notably, experimental evidence indicate that persistent low-dose exposure to arsenic induce healthy lung cells to undergo malignant transformation^[70,71]^. As NRF2 is known to be upregulated by arsenic, a study aimed to determine whether elevated NRF2 levels can promote lung cancer cells’ ability to migrate via a mechanism involving SOX9 transcription factor in A549 and H1299 NSCLC, as well as immortalized lung epithelial BEAS-2B cell lines. To that end, the growth and migration of BEAS-2B NRF2 ^+ / +^ (WT) and NRF2^−/−^ (KO) cell lines were examined after chronic arsenic (termed as As(iii) where cells were cultured with 0.5 μM arsenic for 30 passages (~ 3 months)) exposure to determine the contribution of NRF2 to carcinogenesis. WT cells given chronic As(iii) treatment caused enhanced (~ 5-fold) colony formation. Moreover, in WT cells, the proliferation was augmented, whereas KO cells remained unaffected by chronic As(iii) exposure. Besides, chronic As(iii) treatment to H1299 WT cells caused an enhanced cell migration (~ 2.5-fold faster) compared to untreated control cells, whereas KO cells exhibited no difference in cell migration compared to untreated control cells. Mechanistically, the induction or inhibition of NRF2 caused an increased or decreased expression of SOX9, and SOX9 deletion or NRF2 suppression reduced the growth and metastatic potential of NSCLC cells. Notably, SOX9 deletion reduced the invasion capacity of chronic As(iii) treated BEAS-2B NRF2 ^+ / +^ cells by ~ 66% compared to untreated control cells. These results indicate that lung epithelial cells exposed to chronic arsenic have a considerably lower tendency to invade when SOX9 expression is lost. Additionally, pharmacological suppression of NRF2 by brusatol inhibited the migration and invasion of NSCLC cells, as demonstrated by the reduction in total cell migration and invasion of both H1299 (WT) and H1299 KEAP1^−/−^ (KO) NSCLC cells. Overall, these results indicated that chronic arsenic exposure and loss of KEAP1 function which maintains and activates NRF2 levels, augments NSCLC progression and metastatic potential via NRF2 and SOX9 signaling^[71]^.

Along similar lines, Sun and colleagues conducted a study to investigate the role/mechanisms of AS3MT in peripheral blood lymphocytes of plant workers exposed to arsenic, NSCLC tissues, and A549 cells treated with NaAsO_2_. The urine examination for arsenic traces revealed that tAS (total arsenic), iAs (individual arsenic), MMA (methanearsonic acid), and DMA (dimethylarsinic acid) levels were high in arsenic-exposed subjects. When the correlation between AS3MT mRNA expression and the above-expressed arsenic traces was investigated, the AS3MT expression levels were found to be significantly higher in arsenic-exposed subjects compared to control subjects. Besides, the AS3MT expression level was found to be increased in both the NSCLC tissues and lymph nodes. When A549 cells were treated with NaAsO_2_, it inhibited cell proliferation, while simultaneously, the AS3MT mRNA expression levels were found to be significantly elevated in a concentration-dependent manner. However, AS3MT knockdown attenuated cell proliferation, indicating that AS3MT promotes cell viability and proliferation. Next studies investigated the underlying mechanisms of AS3MT-mediated cell proliferation, and it was found that cyclin E1 and cyclin-dependent kinases 1 (CDKs) were involved in NSCLC cell proliferation. In contrast, the expression of p21, a cyclin-kinase inhibitor and E2F1, a transcription factor was found to be elevated. Overall, these studies indicated that overexpression of AS3MT via long-term arsenic exposure induces NSCLC progression by regulating cell cycle genes^[72]^.

Importantly, there is some evidence demonstrating an association between arsenite methyltransferase (AS3MT, an enzyme involved in arsenic metabolism catalyzing the methylation of arsenic) polymorphism, and arsenic-related cancer risk, including lung cancer^[73,74]^. As inorganic arsenic (iAs) is metabolized mostly in the liver through methylation mechanisms, the rate of breakdown of arsenic varies greatly throughout individuals^[74,75]^. Inter-individual differences in arsenic metabolism activity and vulnerability to arsenic-linked health effects, including bladder and lung malignancies, are influenced by genetic polymorphisms. To determine how intrinsic tumor risk and metabolism of arsenic are related to AS3MT and N-6 adenine-specific DNA methyltransferase 1 (N6AMT1) mutations specifically in susceptible groups (Chile race), de la Rosa and colleagues conducted a study where blood, saliva, and urinary samples were analyzed. Major correlations were documented among urine arsenic metabolite concentrations and AS3MT and N6AMT1 polymorphisms. The AS3MT rs3740393 genotype was associated with greater levels of % monomethylarsonous acid (MMA), an oxidative methylation form of inorganic arsenic (iAs), and % dimethylarsonic acid (DMA), a methylated form of MMA, while the N6AMT1 rs3740390 variant was related to greater amounts of % iAs and % DMA. Furthermore, a link between the % iAs concentrations and the AS3MT rs11191439 polymorphism was noticed. Overall, these studies indicated that there may be a hereditary component linked to the rate of arsenic metabolism, which could affect the population’s susceptibility to arsenic-associated health consequences such as bladder and lung malignancies ^[74]^.

## Conclusion

While a wide range of therapeutic options for NSCLC exists, the ongoing challenges, including drug resistance and cancer recurrence hinder the successful treatment. Thus, it is necessary to devise innovative approaches for overcoming drug resistance and augmenting the efficacy of therapeutic agents for NSCLC treatment. Since drug development is a time-consuming process, drug repurposing provides a promising option for the treatment of NSCLC. To that end, the experimental evidence indicates that arsenic compounds such as As_2_O_3_, arsenic sulfide, and tetra arsenic hexoxide act as promising drug repurposing candidates against NSCLC because of their ability to target oncogenes, including growth factors GAS-1, N-MYC, and Gli1. Importantly, As_2_O_3_ and arsenic sulfide remarkably inhibit the NSCLC stem cells, responsible for chemotherapy resistance (e.g., cisplatin and gefitinib) and tumor recurrence via targeting EGFR pathways, ABC family transporters, and GS-X pumps. Notably, while acute arsenic effects (2 μM-40 μM) are beneficial in managing NSCLC in experimental models, exposure to chronic low dose (100 nM or 0.5 μM) arsenic to normal human bronchial epithelial BEAS-2B cells has been shown to enhance cell proliferation and migration via mechanisms dependent upon the growth/ transcription factors or signaling pathways such as TGFα, EGFR, NRF2, and SOX9. However, such negative effects can be managed by the pharmacological inhibitors of these pathways, which highlights a possible approach to multimodality therapy scenario. Furthermore, individuals with genetic polymorphisms (e.g., in AS3MT enzyme) have also been linked to increased risks of arsenic-induced malignancies, including lung and bladder cancers. Hence arsenic compounds could be explored as a promising drug repurposing approach to overcome drug resistance mechanisms and enhancing the efficacy of therapeutic agents for NSCLC treatment.

## Figures and Tables

**Table 1.** Summary of in vitro and/or in vivo studies.

Authors	Drug(s)	Cell Lines Used	Targets	Findings
Chang *et al*.^[48]^	As_2_O_3_	NCI-H460 NCI-H446	CD133, SOX2, OCT4, Gli-1, GAS-1, N-MYC	As_2_O_3_ decreased CD133, SOX2, and OCT4. Downregulation of Gli-1, GAS-1, N-MYC oncogenes resulted in the inhibition of the hedgehog signaling pathway and the growth of NSCLC cells.
Lee *et al*.^[49]^	As_2_O_3_	NCI-H460	p38-p62 and JNK-Nix	Activation of the JNK pathway induced ROS generation, increased autophagy and apoptosis, and enhanced arsenic sensitivity.
Jiang *et al*.^[58]^	As_2_O_3_	A549	NF-kB, ABCG2	Decreased cell viability and colony formation via upregulation of ABCG1 and NF-κB activation.
	As_2_O_3_ ± PDTC and novobiocin			Potentiated As2O3 cytotoxicity and increased apoptosis via modulating NF-kB expression.
Suzuki *et* al.^[19]^	As_2_O_3_	PC-9/CDDP PC-14/DDP	GS-X pumps and ABC family transporters	Induction of arsenic sensitivity resulted via downregulation of GS-X pumps and ABC transporter family activity.
Yang et al.^[47]^	As_2_O_3_	NCI-H460 NCI-H466 A549	VEGFR-A, VEGFR-2, HIF-Iα, DII4-Notch pathway	Tumor suppression via downregulation of VEGF-A and VEGFR-2 expression in both NSCLC and SCLC. Decreased tumor angiogenesis by downregulation of HIF-1α, and Dll4-Notch pathway in both NSCLC and SCLC.
Mao *et* al.^[51]^	As_2_O_3_ As_2_O_3_ + Gefitinib As_2_O_3_ + NH4CI + BafAl (bafilomycin A1)	NCI-H1975 HCC827	EGFR	Induction of autophagy and apoptosis. Decreased cell proliferation and tumor growth in NOD-SCID mice. EGFR degradation, induction of autophagy, decreased tumor growth and inhibition of gefitinib drug resistance.
Tian *et al*.^[39]^	As_4_S_4_ + DDP As_4_S_4_ + DDP	A549/DDP H1299/DDP	p53, PD-L1	Downregulation of PD-L1 expression by the induction of p53. No effect
Lee *et al*.^[50]^	As_4_O_6_ (PR) + Oldenlandia diffusa, Salvia miltiorrhiza (OS), and PROS	A549, NH460, HCT-116, 786-O, PC-3, DU145, YD-8, H1299, and HUVEC	STAT3, COX-2, ERK, Src, AKT, SOCS-1, VEGFR2/SRC/STAT3 axis	Induction of apoptosis and G1- and S-phase cell cycle arrest by the downregulation of Bcl- 2, and activation of ERK, Src, AKT, COX-2, SOCS-1 as well as disruption of the interaction between STAT3-CDK2 and VEGF-STAT3 pathways (i.e., STAT3/VEGF/CDK2 axis. Decreased tumor growth via induction of apoptosis.

**Table 2.** Summary of studies with long-term arsenic exposure.

Authors	Drug(s)	Cell Lines Used	Targets	Findings
Kim *et al*.^[69]^	NaAsO_2_	BEAS-2B	TGFα, EGFR	Chronic arsenic activates TGFα that causes over-expression of EGFR and promotes cell migration.
Schmidlin *et al*.^[71]^	Arsenic	BEAS-2B NRF2 ^+ / +^ (WT) NRF2^−/−^ (KO), H1299 WT, H1299 KEAP1^−/−^	SOX-9, NRF2	Chronic arsenic exposure augments NSCLC progression and metastatic potential via targeting NRF2 and SOX9 signaling.
Sun *et al*.^[72]^	NaAsO_2_	A549	AS3MT	Prolonged arsenic-mediated AS3MT overexpression and induces NSCLC progression via upregulating cell cycle genes.

## Abbreviations

ABC: adenosine triphosphate (ATP)-binding cassette
ADCs: antibody-drug conjugates
ALK: anaplastic lymphoma kinase
APL: acute promyelocytic leukemia
As_2_O_3_: arsenic trioxide
AsIII(GS)3: tri gamma-glutamyl cysteinyl glycine thioarsenite
CDDP: cisplatin
CTLA-4: cytotoxic T-lymphocyte-associated protein 4
EGFR: epidermal growth factor receptor
GAS1: growth arrest specific-1 gene
Gli1: glioma-associated oncogene-1
HGF: hepatocyte growth factor
HGFR: hepatocyte growth factor receptor
ICI: immune checkpoint immunotherapy
JNK: c-Jun N-terminal kinase
MAPK: mitogen-activated protein kinases
MET: mesenchymal-epithelial transition tyrosine kinase receptor
NaAsO_2_: sodium arsenite
NAC: N-acetylcysteine
NF-κB: nuclear factor kappa B
N-myc: N-myc proto-oncogene protein
NSCLC: Non-small cell lung cancer
Oct4: octamer-binding transcription factor 4
PD-1: programmed death receptor 1
PFS: progression-free survival
RFA: radiofrequency ablation
ROS: reactive oxygen species
ROS1: receptor tyrosine kinase
RT: radiation therapy
SCLC: small cell lung cancer
SoX2: SRY-box transcription factor 2
TKI: tyrosine kinase inhibitor
TNF: α, tumor necrosis factor α
